# Regulation of Anthocyanin Accumulation in Tomato *Solanum lycopersicum* L. by Exogenous Synthetic dsRNA Targeting Different Regions of *SlTRY* Gene

**DOI:** 10.3390/plants13172489

**Published:** 2024-09-05

**Authors:** Andrey R. Suprun, Artem Yu. Manyakhin, Evgeniya V. Trubetskaya, Konstantin V. Kiselev, Alexandra S. Dubrovina

**Affiliations:** Federal Scientific Center of the East Asia Terrestrial Biodiversity, Far Eastern Branch of the Russian Academy of Sciences, 690022 Vladivostok, Russiadubrovina@biosoil.ru (A.S.D.)

**Keywords:** *Solanum lycopersicum*, exogenous dsRNA, gene silencing, RNA interference, plant foliar treatment, plant gene regulation, tomato, transcription factors

## Abstract

RNA interference (RNAi) is a regulatory and protective mechanism that plays a crucial role in the growth, development, and control of plant responses to pathogens and abiotic stresses. In spray-induced gene silencing (SIGS), exogenous double-stranded RNAs (dsRNA) are used to efficiently regulate target genes via plant surface treatment. In this study, we aimed to evaluate the effect of specific exogenous dsRNAs on silencing different regions (promoter, protein-coding and intron) of the target *SlTRY* tomato gene, encoding an R3-type MYB repressor of anthocyanin biosynthesis. We also assessed the impact of targeting different *SlTRY* regions on the expression of genes involved in anthocyanin and flavonoid biosynthesis. This study demonstrated the critical importance of selecting the appropriate gene target region for dsRNA action. The highest inhibition of the *SlTRY* gene expression and activation of anthocyanin biosynthesis was achieved by dsRNA complementary to the protein-coding region of *SlTRY* gene, compared with dsRNAs targeting the *SlTRY* promoter or intron regions. Silencing the *SlTRY* gene increased the content of anthocyanins and boosted levels of other substances in the phenylpropanoid pathway, such as caffeoyl putrescine, chlorogenic acid, ferulic acid glucoside, feruloyl quinic acid, and rutin. This study is the first to examine the effects of four different dsRNAs targeting various regions of the *SlTRY* gene, an important negative regulator of anthocyanin biosynthesis.

## 1. Introduction

Tomato (*Solanum lycopersicum* L.) is one of the most widely grown and consumed vegetable crops in the world [1]. Tomato is also widely used in molecular biology as a model object, including for studying the mechanism of anthocyanin biosynthesis [2,3,4]. Anthocyanins are a class of flavonoids formed by the phenylpropanoid biochemical pathway in plants. They have a common basal structure consisting of two aromatic benzene rings separated by an oxygen-containing heterocycle consisting of three carbon atoms [5]. Glycosylated forms of anthocyanins are stored in the vacuoles of plant cells, contributing to the diverse colors observed in flowers and fruits, such as red, blue, and purple. These vibrant hues play a crucial role in attracting pollinators and animals, which aids in both pollination and seed dispersal [2,5]. In plant tissues, anthocyanins help protect plants from a variety of biotic and abiotic stressors, including those caused by insects, pathogenic fungi and bacteria, as well as drought, UV radiation and low temperatures [6,7,8]. Notably, anthocyanins are extremely beneficial for human health due to their antioxidant activity and ability to influence signaling pathways in animal cells [2]. Studies have shown that eating foods that reach these metabolites reduces the risk of cancer, coronary heart disease, atherosclerosis, obesity and diabetes [9,10,11,12].

**Figure 1:**
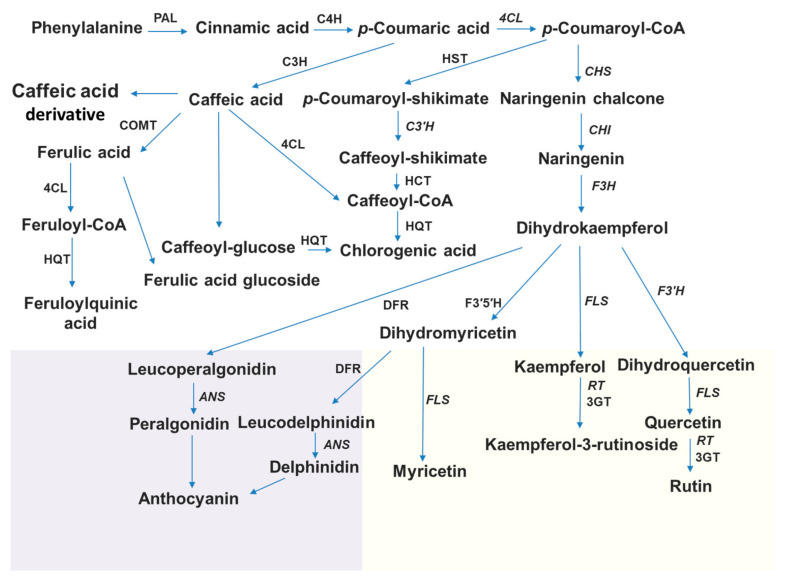
Schematic phenylpropanoid pathway in tomato. Phenylalanine-ammonia-lyase (PAL), cinnamate 4-hydroxylase (C4H), 4-coumarate-CoA ligase (4CL), chalcone synthase (SlCHS1, SlCHS2), chalcone isomerase (CHI), flavanone-3-hydroxylase (F3H), flavonoid-3′-hydroxylase (F3´H), flavonol synthase (FLS), flavonoid-3′-5′-hydroxylase (F3′5′H), dihydroflavonol reductase (DFR), anthocyanidin synthase (ANS), flavonoid 3-O-glucoside-rhamnosyltransferase (RT), flavonoid-3-O-glucosyltransferase (3GT), 4-coumaroyl shikimate/quinate 3′-hydroxylase (C3′H), cinnamoyl-CoA shikimate/quinate transferase (HCT), p-coumaroyl ester 3-hydroxylase (C3H), hydroxycinnamoyl-CoA quinate transferase (HQT), hydroxycinnamoyl-CoA shikimate/quinate hydroxycinnamoyltransferase (HCT), caffeate O-methyltransferase (COMT). Lilac color represents the biosynthesis of anthocyanins, and yellow color represents the biosynthesis of flavonols and flavonol glycosides. Genes analyzed in this work are shown in italics. Based on the article of Rigano et al. [30].

Anthocyanins, the pigments responsible for the vibrant reds, purples, and blues in fruits, vegetables, and flowers, are produced through a complex biochemical pathway involving a series of enzymatic reactions. This process, known as anthocyanin biosynthesis, begins with the amino acid phenylalanine, which undergoes a series of transformations catalyzed by specific enzymes, such as phenylalanine ammonia lyase (PAL), 4-coumaryl–CoA ligase (4CL), chalcone synthase (CHS), chalcone isomerase (CHI), flavanone 3-hydroxylase (F3H), flavonoid 3′-hydroxylase (F3′H), flavonoid 3′5′-hydroxylase (F3′5′H), dihydroflavonol-4-reductase (DFR), anthocyanidin synthase (ANS), flavonol 3-glucosyltransferase (3GT) and rhamnosyltransferase (RT) [13] (Figure 1). The expression of early biosynthetic genes (EBG) (CHS, CHI, F3H) is modulated by transcription factors of the R2R3-MYB subgroup. Late biosynthetic genes (LBGs) (DFR, ANS, UFGT) are regulated by three transcription factors known as the ternary MBW complex (R2R3-MYB, bHLH and WD40) [13,14]. MYB transcription factors play a crucial role in regulating the production of anthocyanins and act as molecular switches, either activating or repressing the expression of genes involved in anthocyanin biosynthesis. To exert their influence, MYB factors team up with other protein partners, namely bHLH (basic helix–loop–helix) and WD40 factors forming the MBW complex [15]. In recent years, an increasing number of regulatory proteins have been characterized. These also include negative regulators, either R2R3-MYB or R3-MYB proteins, which have one or two MYB domain repeats, respectively, that can disrupt the activity of the MBW complex [14,16,17]. Tomato plants carrying a mutation in the *SlMYBATV* gene showed an increased expression of biosynthetic genes and accumulated increased amounts of anthocyanins [16]. MYBATV has also been shown to interact with tomato bHLH factors involved in MBW complexes, resulting in the disruption of its activity and suppression of anthocyanin production. Colanero et al. [17], using a genome-wide screen, identified two genes of transcription factors (TFs) (*SlTRY* and *SlMYBATV*) as R3-type MYB repressors, and four genes of TFs (*SlMYB3*, *SlMYB7*, *SlMYB32* and *SlMYB76*) were identified as R2R3-type MYB repressors. The overexpression of *SlTRY* in Arabidopsis was also shown to result in decreased anthocyanin accumulation [18]. The involvement of *SlTRY* as a negative regulator is also supported by our previous study, in which the inhibition of this gene via the RNA interference mechanism resulted in increased accumulation of anthocyanins in tomato [4].

RNA interference is a regulatory and protective mechanism involved in growth and development processes, as well as in the control of plant responses to pathogens or abiotic stresses [19]. RNA interference is known to be involved in plant responses to unwanted nucleic acids and transposons, and in regulating the expression of endogenous protein-coding genes. The process of RNA interference in a plant begins with exogenous dsRNA entering the cell and binding to DCL ribonuclease, which cleaves it into small fragments 20–25 bp long, with two unpaired bases at the 5′ and 3′ ends. The fragments interact with the RISC complex, which cleaves one of the RNA strands. Preference is given to the fragment whose 5′ end is less tightly conjugated. The second RNA strand is cleaved by endonucleases. The resulting complex moves around the cell in search of homologous messenger RNA. Having found it, the AGO protein from the RISC complex cuts the messenger RNA [19,20].

Increasingly, the RNA interference mechanism is used as a tool for molecular biology research to regulate gene expression in order to study their functions and improve plant quality [21]. SIGS allows the use of exogenous RNAs for the simple and efficient regulation of target genes via the RNAi mechanism [21,22]. This method allows us to combat fungal or bacterial pathogens and viral diseases, and also to regulate the expression of the plants’ own genes [4,22,23,24]. External treatment of plants with dsRNA complementary to target plant genes resulted in a decreased expression of target genes, namely the *EPSPS* gene (3-phosphate synthase gene) in the leaves of tobacco and amaranth [25]; Myb1 gene in orchid flower buds [26]; *Mob1A*, *WRKY23* and actin genes in *Arabidopsis thaliana* [27]; and *LBDIf7* and *GST40* genes in grapevine [23,28]. The research findings indicate that the external treatment of plant surfaces with gene-specific dsRNA resulted in alterations to the phenotype and biochemical processes, thereby enhancing the plants’ resistance to fungal infection and improving their resilience to abiotic stress [23,28]. In our previous study, we showed that the efficiency of silencing the *A. thaliana NPTII* transgene using exogenous dsRNA is affected by factors such as plant treatment time, soil moisture, plant age, the method of applying dsRNA to the plant surface and various abiotic factors. However, there is currently no specific information on the effect of the region of the target gene to which the dsRNA is complementary on the efficiency of silencing [29].

The objective of the present study was to evaluate the effect of specific exogenous dsRNA on different regions of the tomato anthocyanin biosynthesis negative regulator gene *SlTRY* at the mRNA level. Additionally, we assessed the impact on the expression of several genes involved in anthocyanin and other flavonoid biosynthesis, including 4-coumarate-CoA ligase (4CL), chalcone synthase (SlCHS1, SlCHS2), chalcone isomerase (CHI), flavanone-3-hydroxylase (F3H), flavonoid-3′-hydroxylase (F3′H), flavonol synthase (FLS), anthocyanidin synthase (ANS), flavonoid 3-O-glucoside-rhamnosyltransferase (RT) and 4-coumaroyl shikimate/quinate 3′-hydroxylase (C3′H). We also investigated the effect of dsRNA-TRY on the content and profile of anthocyanins and other secondary metabolites. Through this comprehensive analysis, we aimed to better understand the role of *SlTRY*, the regulatory mechanisms influencing anthocyanin biosynthesis and the broader flavonoid pathway in tomato plants.

## 2. Results

### 2.1. Exogenous dsRNAs Downregulate mRNA Levels SlTRY Transcription Factors

**Figure 2:**
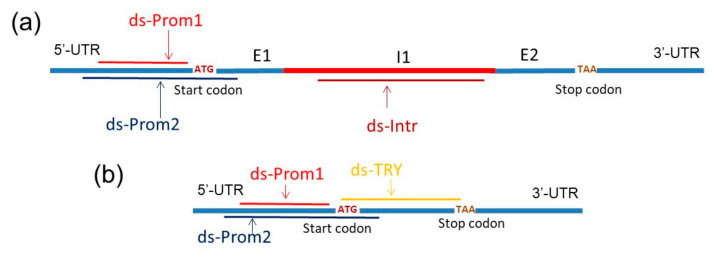
Schematic representation of the dsRNAs positions on the *SlTRY* gene. (**a**) Positions of dsRNA-Prom1 (308 bp), dsRNA-Prom2 (386 bp) and dsRNA-Intron (483 bp) on pre-mRNA; (**b**) positions of dsRNA-Prom1 (308 bp), dsRNA-Prom2 (386 bp) and dsRNA-TRY (285 bp) on mRNA. E1—Exon 1, I1—Intron, E2—Exon 2.

We obtained in vitro synthesized dsRNAs complementary to different regions of the *SlTRY* gene, which negatively regulates anthocyanin biosynthesis in tomato leaves. First, dsRNA was generated to target solely the promoter region of the SlTRY gene, designated as dsRNA-Prom1 (Figure 2a,b). Next, we synthesized dsRNA that targeted both the promoter and the beginning of the protein coding region, designated as dsRNA-Prom2 (Figure 2a,b). dsRNA-TRY targeted the entire coding sequence (Figure 2b). It is known that the *SlTRY* gene contains one intron, which is removed during splicing. We were interested in whether dsRNA targeting an intron is able to initiate the RNA interference of immature mRNA, namely pre-mRNA. For this purpose, we obtained dsRNA targeting the intron of the *SlTRY* gene, designated as dsRNA-Intron (Figure 2a).

**Figure 3:**
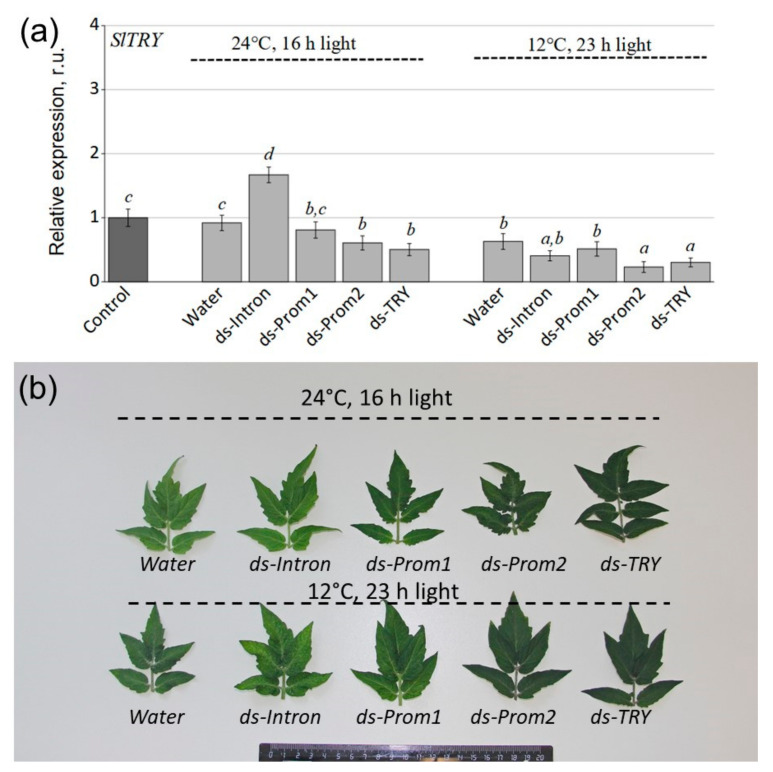
(**a**) Relative fold change in *Solanum lycopersicum* mRNA levels after treatment with dsRNA compared to untreated plants. (**b**) Photograph of leaves on day 7 after treatment of plants with water and dsRNAs under normal conditions and conditions inducing anthocyanin accumulation. Control—average gene expression before treatment; Water—*S. lycopersicum* treated with sterile water; ds-Prom1—S. lycopersicum treated with dsRNA-Prom1; ds-Prom2—*S. lycopersicum* treated with dsRNA-Prom2; ds-Intron—*S. lycopersicum* treated with dsRNA-Intron; dsTRY—*S. lycopersicum* treated with dsRNA-TRY. Data are presented as mean ± standard error. The means in each figure with the same letters were not different from each other according to one-way analysis of variance (ANOVA) followed by Tukey’s multiple comparison test.

Then, we analyzed whether the exogenous application of our prepared dsRNA-Prom1, dsRNA-Prom2, dsRNA-TRY and dsRNA-Intron double-stranded RNAs to the leaf surface of five-week-old *S. lycopersicum* could result in any changes in the mRNA transcript levels of the *SlTRY* gene compared to water-treated controls seven days after dsRNA treatment (Figure 3). Since anthocyanin production and the expression of anthocyanin biosynthesis genes in *S. lycopersicum* were low under standard cultivation conditions, we divided the treated plants into two groups for post-treatment incubation. The first group consisted of plants grown under control conditions (22 °C, 16 h light), while the second group was grown under anthocyanin-inducing conditions (12 °C, 23 h light) for seven days. This was carried out to induce the expression of anthocyanin biosynthetic genes and the accumulation of anthocyanins in tomato leaves. Additionally, we aimed to analyze the effects of dsRNA on these processes.

qRT–PCR analysis showed that the mRNA level of the *SlTRY* gene was significantly lower after leaf treatment with dsRNA-Prom2 and dsRNA-TRY under both standard and anthocyanin-inducing conditions (Figure 3a). The treatment of plants with dsRNA-Prom1 and dsRNA-Intron did not significantly decrease the expression of the *SlTRY* gene. Moreover, dsRNA-Intron treatment increased the expression of the *SlTRY* gene compared to the control, but this effect was observed only under standard conditions (Figure 3a). 

dsRNA-Intron treatment can cause an increase in *SlTRY* gene expression, potentially through an intron-mediated expression enhancement mechanism [31]. The fact that this effect was only observed under standard conditions should suggest that stress conditions may negate the regulatory effects of dsRNA-Intron treatment, but this requires further investigation. Furthermore, the changes in *SlTRY* gene expression following dsRNA treatments corresponded with changes in the color intensity of *S. lycopersicum* leaves. The darkest tomato leaves were observed after treatment with dsRNA-Prom2 and dsRNA-TRY under both standard and anthocyanin-inducing conditions (Figure 3b). These results suggest that the efficient silencing of a plant target gene requires dsRNA to target a protein-coding region of at least 50 base pairs in length (like dsRNA-Prom2).

### 2.2. Exogenous dsRNAs Upregulate mRNA Levels of Phenylpropanoid Biosynthesis Pathway Genes

**Figure 4:**
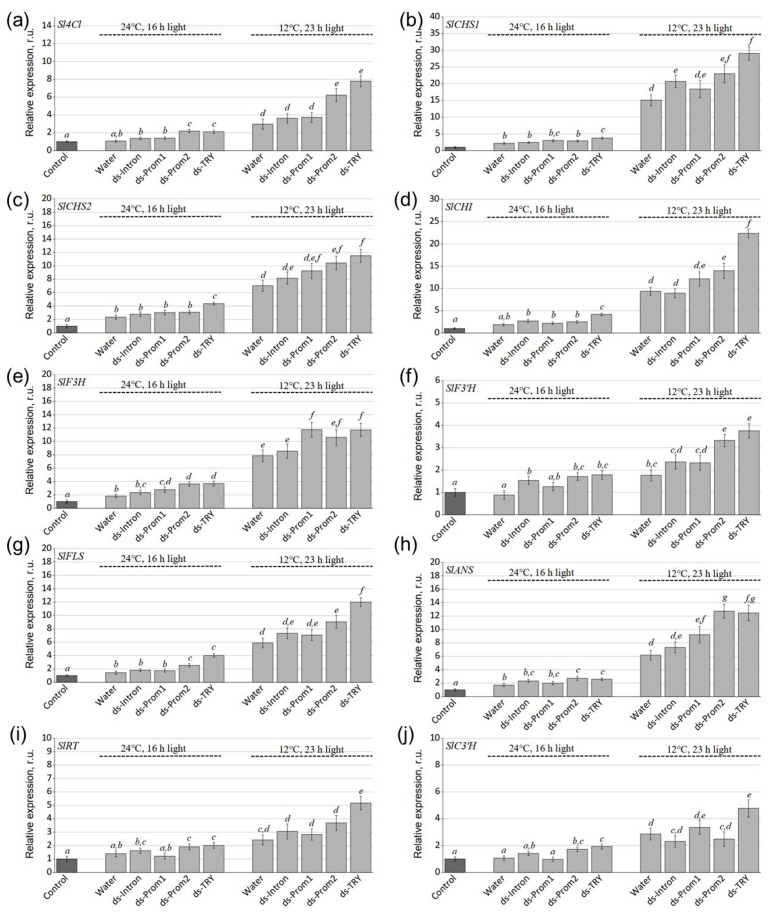
Relative fold changes in (**a**) *Sl4CL*, (**b**) *SlCHS1*, (**c**) *SlCHS2*, (**d**) *SlCHI*, (**e**) *SlF3H*, (**f**) *SlF3′H*, (**g**) *SlFLS*, (**h**) *SlANS*, (**i**) *SlRT* and (**j**) *SlC3′H* mRNA levels after dsRNA treatments of *Solanum lycopersicum* compared to untreated plants. Control—average gene expression before treatment; Water—*S. lycopersicum* treated with sterile water; ds-Prom1—*S. lycopersicum* treated with dsRNA-Prom1; ds-Prom2—*S. lycopersicum* treated with dsRNA-Prom2; ds-Intron—*S. lycopersicum* treated with dsRNA-Intron; dsTRY—*S. lycopersicum* treated with dsRNA-TRY. Data are presented as mean ± standard error. The means in each figure with the same letters were not different from each other according to one-way analysis of variance (ANOVA) followed by Tukey’s multiple comparison test.

Then, we studied the effect of exogenous Prom1-, Prom2-, Intron- and SlTRY-dsRNAs on the expression of phenylpropanoid biosynthesis pathway genes, such as 4-coumaroyl–CoA ligase (*Sl4CL*), chalcone synthase (*SlCHS1* and *SlCHS2*), chalcone isomerase (*SlCHI*), flavanone 3-hydroxylase (*SlF3H*), flavonoid 3′-hydroxylase (*SlF3′H*), flavonol synthase (*SlFLS*), anthocyanidin synthase (*SlANS*), rhamnosyltransferase (*SlRT*) and 4-coumaroyl shikimate/quinate 3′-hydroxylase (*SlC3′H*) (Figure 4). These genes are key enzymes in the early and late biosynthesis of flavonoids, including anthocyanins and flavonols [13].

The analysis revealed a significant increase in the expression of all analyzed genes in *S. lycopersicum* grown under anthocyanin-inducing conditions compared to control conditions (Figure 4). This suggests that stress conditions activate the phenylpropanoid pathway. The expression levels of *Sl4CL*, *SlCHS1*, *SlCHS2*, *SlCHI*, *SlF3H*, *SlF3′H*, *SlFLS*, *SlANS*, *SlRT* and *SlC3′H* were notably higher (1.4–2.9 and 1.5–2.6 times under normal conditions and conditions inducing anthocyanin accumulation) in dsRNA-TRY-treated plants than in water-treated *S. lycopersicum* plants (Figure 4). Treatment with dsRNA-Prom2 also significantly upregulated the expression of most of the studied genes by 1.4 to 2.1 times compared to water-treated plants under anthocyanin-inducing conditions, with the exception of the *SlF3H*, *SlRT* and *SlC3′H* genes (Figure 4). Under control conditions, dsRNA-Prom2 also led to gene activation by 1.5 to 2 times compared to water-treated plants, though no significant activation was observed for *SlCHS1*, *SlCHS2* and *SlCHI* genes (Figure 4b–d). 

Interestingly, using dsRNA-Prom1 targeting the promoter of the *SlTRY* gene resulted in a 1.5-fold increase in *SlF3H* gene expression compared to water-treated plants under both conditions, and an increase in *SlANS* gene expression under anthocyanin-inducing conditions (Figure 4e,h). However, the application of exogenous dsRNA-Intron did not lead to significant changes in the expression of phenylpropanoid pathway genes under either condition (Figure 4).

### 2.3. Exogenous dsRNA Upregulates Secondary Metabolism

**Figure 5:**
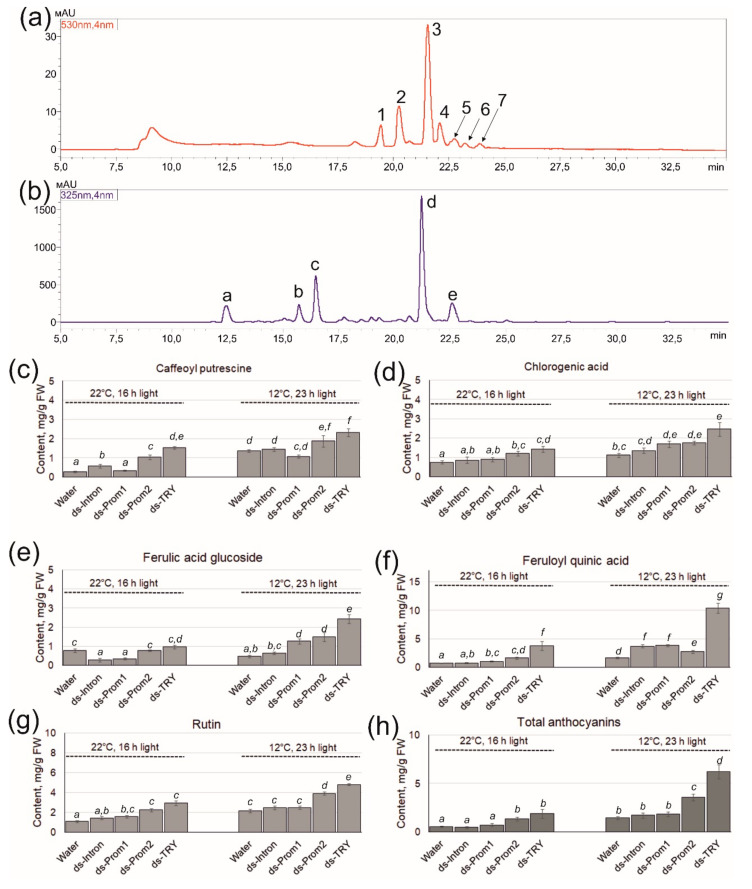
Content of secondary metabolites in the leaves of *Solanum lycopersicum* seven days after treatment with the dsRNAs under control conditions and anthocyanin modulatory conditions. (**a**) A representative HPLC-UV profile (530 nm) of anthocyanins; (**b**) a representative HPLC-UV profile (325 nm) of other secondary metabolites; (**c**) caffeoyl putrescine content; (**d**) chlorogenic acid content; (**e**) ferulic acid glucoside content; (**f**) feruloyl quinic acid content; (**g**) rutin content; (**h**) total anthocyanin content. (1) Petunidin-3.5-O-diglucoside; (2) petunidin-3-(caffeoyl)-rutinoside-5-glucoside; (3) petunidin-3-(p-coumaroyl)-rutinoside-5-glucoside; (4) delphinidin-3-O-(6″-O-p-coumaroyl)-glucoside; (5) delphinidin-3-O-glucoside; (6) malvidin-3-(p-coumaroyl)-rutinoside-5-glucoside; (7) cyanidin-3-O-(6″-O-p-coumaroyl)-glucoside; (a) caffeoyl putrescine; (b) chlorogenic acid; (c) ferulic acid glucoside; (d) feruloyl quinic acid; (e) rutin. *S. lycopersicum* was grown under control or anthocyanin-inducing conditions. Data are presented as mean ± standard error. The means in each figure with the same letters were not different from each other according to one-way analysis of variance (ANOVA) followed by Tukey’s multiple comparison test.

We further studied the effects of exogenous Prom1-, Prom2-, Intron-, and SlTRY-dsRNAs on secondary metabolism using HPLC–mass spectrometry (HPLC-MS). According to HPLC-MS analysis, seven anthocyanin compounds were shown to be present in *S. lycopersicum* leaves, namely petunidin-3.5-O-diglucoside (1), petunidin-3-(caffeoyl)-rutinoside-5-glucoside (2), petunidin-3-(p-coumaroyl)-rutinoside-5-glucoside (3), delphinidin-3-O-(6″-O-p-coumaroyl)-glucoside (4), delphinidin-3-O-glucoside (5), malvidin-3-(p-coumaroyl)-rutinoside-5-glucoside (6) and cyanidin-3-O-(6″-O-p-coumaroyl)-glucoside (7) (Figure 5a, Table S2). It is possible that other anthocyanins were also present in the analyzed tissues of *S. lycopersicum*, but in trace amounts. Moreover, we have shown the presence of five substances in the leaves of *S. lycopersicum* that are related to caffeic acid derivatives and flavonols, namely caffeoyl putrescine (a), chlorogenic acid (b), ferulic acid glucoside (c), feruloyl quinic acid (d) and rutin (e) (Figure 5a–g). In addition, the content of most of the analyzed compounds was higher in plants grown under stress conditions (Figure 5c–h).

According to HPLC-MS analysis, this dsRNA-SlTRY-induced decrease in the *SlTRY* gene expression and increase in anthocyanin biosynthetic gene expression correlated with a significant increase in anthocyanin accumulation, reaching 2.2 and 6.2 mg/g FW under control and anthocyanin accumulation-inducing conditions, respectively (Figure 5h). A significant increase in tissue content was also observed after dsRNA-Prom2 treatment, but at lower levels than after dsRNA-SlTRY treatment, namely 1.3 and 3.6 mg/g FW under control and anthocyanin accumulation-inducing conditions, respectively. Importantly, the increase in anthocyanin content was achieved by petunidin-3-(caffeoyl)-rutinoside-5-glucoside and petunidin-3-(p-coumaroyl)-rutinoside-5-glucoside, which accounted for up to 85% of the total anthocyanins. The application of dsRNA-Intron and dsRNA-Prom1 did not lead to a significant increase in anthocyanin content compared to water-treated plants under both types of conditions, which is generally consistent with the expression of biosynthetic genes (Figure 5h).

Interestingly, the application of exogenous dsRNA-SlTRY and dsRNA-Prom2 affected not only the increase in the content of anthocyanins but also other secondary metabolites such as caffeoyl putrescine, chlorogenic acid, ferulic acid glucoside, feruloyl quinic acid and rutin, in contrast to dsRNA-Intron and dsRNA-Prom1, which did not have a significant effect (Figure 5a–g). The content of caffeoyl putrescine, which is involved in plant defense against insects [32], after the application of dsRNA-SlTRY, increased by 5.8 and 2 times in control and stress conditions, respectively, compared to water-treated plants (Figure 5c). The amount of chlorogenic acid increased by 2 times after the application of dsRNA-SlTRY and by 1.5 times after the application of dsRNA-Prom2 compared to the control in both types of conditions (Figure 5c). An increase in the content of ferulic acid glucoside was noted only under stress conditions up to 5.2 times after the application of dsRNA-SlTRY (Figure 5e). A pronounced positive effect of dsRNA-SlTRY application was exerted on the content of feruloyl quinic acid, namely by 5 and 6.2 times in control and stress conditions, respectively, compared to the control (Figure 5f). The amount of rutin also increased by 2.2 times in both types of conditions (Figure 5g). Our findings, which reveal a rise in the content of the analyzed substances as a result of dsRNA application, align well with the gene expression data. It can be assumed that the activation of early biosynthetic genes of the phenylpropanoid pathway, as a result of the inhibition of the negative regulator of biosynthesis TRY, has a positive effect on the entire secondary metabolism, and not only on the biosynthesis of anthocyanins.

## 3. Discussion

In today’s world, it is essential to create innovative and effective methods to enhance and safeguard plant productivity through safe and eco-friendly technologies. The growing human population and negative impacts of environmental stresses necessitate the development of new molecular tools to improve and protect crops without modifying their genomes. Recently, a novel method known as SIGS has gained traction for directing plant traits in the desired way [19,33,34]. SIGS technology enables the protection of plants from insect pests, fungal infections, and viral pathogens by manipulating the expression of key genes in either the plants themselves or the pathogens [35,36,37]. For plant protection, foliar spray application is one of the most effective methods for delivering dsRNA in terms of cost, time, and labor. However, many questions remain about the effectiveness of SIGS application compared to traditional plant protection methods such as chemical treatments.

In particular, there is currently limited knowledge about the ability to regulate plant gene expression by applying dsRNA or siRNA to the plant surface. Research has demonstrated that treating plants with dsRNA targeting endogenous plant genes can suppress the mRNA levels of these genes. For instance, the transcript levels of the *EPSPS* gene were reduced in tobacco and amaranth leaves [25], and in orchid buds, the *Myb1* gene was suppressed [26]. The application of in vitro synthesized dsRNA complementary to the *SlIAA9* and *SlAGL6* genes of tomato (*S. lycopersicum* cv UC82) resulted in the decreased expression of these genes and increased ovary size during flowering [34]. Treating grapevines with specific dsRNA targeting the *LBDIf7* gene resulted in the silencing of the *VviLBDIf7* gene and led to a decrease in infection and sporulation of *Plasmopara viticola*, a pathogenic organism affecting grapevines [23]. We have also previously shown that exogenous dsRNA targeting the *AtCHS* gene, as well as two important genes encoding transcriptional repressors of anthocyanin biosynthesis in Arabidopsis, AtMYBL2 and AtANAC032, can effectively influence gene expression [4]. Thus, the potential of using exogenous treatment of plants with targeted dsRNAs to modify plant phenotypes and biochemical responses has been demonstrated. This includes altering flower morphology, enhancing resistance to fungi and increasing tolerance to drought stress.

It is well established that the efficiency of gene silencing via dsRNA depends on various factors. These include the specific plant species involved, the selection of the dsRNA target, the design of the dsRNA itself, the method and timing of delivery to the plant surface and the prevailing environmental conditions. Each of these factors can significantly influence the success and effectiveness of the RNA interference (RNAi) process in plants [29]. While bioinformatics offers numerous tools for the design, analysis and evaluation of small RNA (sRNA) agents, the impact of dsRNA design on its processing and the efficiency of target gene silencing remains poorly understood. Further research is needed to elucidate how specific design parameters influence the effectiveness of dsRNA in achieving desired gene silencing outcomes. [35]. Höfle et al. [35] demonstrated that the reduction in SIGS-mediated disease resistance in *Fusarium graminearum* was significantly correlated with the length of the dsRNA construct that was sprayed. Longer dsRNA constructs were more effective in conferring disease resistance. It has also been shown that using high concentrations of dsRNA targeting the EGFP and NPTII genes of *A. thaliana* causes a stronger silencing effect [38]. As of now, there is a lack of studies specifically assessing the influence of the region of the transcript targeted by dsRNA on the efficacy of RNA interference in plants. Research in this area could provide valuable insights into whether certain regions of the transcript are more conducive to effective gene silencing and could potentially optimize dsRNA design strategies for enhanced RNAi efficiency.

This study was the first to examine the effects of four different dsRNAs targeting various regions of the *SlTRY* gene, an important negative regulator of anthocyanin biosynthesis. Although anthocyanins are present in a variety of plant species, their quantities are often limited or entirely absent in many plants due to restricted activity of the flavonoid biosynthesis pathway [39]. Our aim was to study the effect of dsRNA specific to different regions of immature and mature *SlTRY* transcripts on the silencing of this gene. We designed and produced dsRNA targeting the promoter part of the gene, the promoter and protein-coding region, the protein-coding region only and the intron. We found that the greatest activation of anthocyanin biosynthesis was achieved using dsRNA complementary to the protein-coding region of *SlTRY*. We also obtained interesting data indicating that dsRNA-Intron treatment increased the expression of the *SlTRY* gene compared to the control. However, this effect was observed only under standard conditions. We hypothesize that exogenous treatment with dsRNA-Intron may induce an increase in *SlTRY* gene expression through an intron-mediated enhancement mechanism [31]. It is possible that stress conditions could negate the regulatory effects of dsRNA-Intron enhancement, as abiotic stresses have been shown to affect the efficiency of dsRNA treatment [29].

Using the SIGS approach, we increased the total amount of anthocyanins in tomato leaves by 3.5 and 4.4 times under control and anthocyanin accumulation-inducing conditions, respectively, compared to water-treated plants. This convincingly demonstrates the role of the R3-MYB TRY protein as a competitive inhibitor of MBW complexes [14,40]. TFs are thought to exert their repressive function through competition, known as passive repression, for bHLH partners with R2R3-MYB factors that activate anthocyanin synthesis. This competitive interaction can limit the availability of bHLH partners for activator MYB proteins, thereby reducing the expression of genes involved in the anthocyanin biosynthesis pathway [8,14,41]. The most studied genes are *CAPRICE* (*CPC*) and *TRIPTYCHON* (*TRY*) in Arabidopsis, which mainly inhibit MBW complexes involved in the formation of trichomes and root hairs but also affect flavonoid production [17,18]. A CPC mutant in Arabidopsis has been reported to have elevated anthocyanin levels, suggesting that CPC may normally contribute to anthocyanin regulation [40]. 

Interestingly, we found that silencing the *SlTRY* gene not only increases the content of anthocyanins but also other substances in the phenylpropanoid pathway, such as caffeoyl putrescine, chlorogenic acid, ferulic acid glucoside, feruloyl quinic acid and rutin. These results align with a study demonstrating that the overexpression of *VvMTBC2L2*, a *Vitis vinifera* transcription factor, leads to a significant downregulation of genes involved in flavonoid production [42]. Nakatsuka et al. employed a chimeric RNAi construct to downregulate two genes, flavonol synthase (*FLS*) and flavonoid 3′-hydroxylase (*F3′H*), along with the expression of the gerbera dihydroflavonol 4-reductase (*DFR*) gene, resulting in abundant accumulation of flavonol derivatives in the form of colorless flavonoids and colored anthocyanins [43]. 

In summary, this study demonstrated the critical importance of selecting the appropriate gene target region for dsRNA action. Targeting the protein coding sequence of a gene maximally silenced its expression, highlighting dsRNA as a powerful tool for rapidly investigating gene function in plant research. Moreover, this approach holds promise in plant biotechnology for enhancing the production of bioactive compounds.

## 4. Materials and Methods

### 4.1. Plant Material and Growth Conditions

Seeds of the wild-type tomato cultivar Micro-Tom (*S. lycopersicum*) were sourced from the Laboratory of Biotechnology at the Federal Scientific Center of the East Asia Terrestrial Biodiversity, Vladivostok, Russia. The seeds underwent vapor-phase sterilization as described in [44] and were subsequently planted in pots (9 cm × 9 cm) containing 200 g of commercially available nutrient-rich soil, which was well irrigated from the bottom with filtered water. The plants were cultivated in a growth chamber (Sanyo MLR-352, Panasonic, Osaka, Japan) under a light intensity of approximately 120 μmol·m^−2^·s^−1^ with a 16 h daily light cycle at 22 °C for four weeks before receiving dsRNA treatments. Following the dsRNA treatments, the *S. lycopersicum* plants were further incubated for seven days either under control conditions (22 °C, 16 h light cycle) or under anthocyanin-inducing conditions (12 °C, 23 h light cycle) in a growth chamber (KS-200, Smolenskoye SKTB SPU, Smolensk, Russia), without additional irrigation, to promote anthocyanin accumulation.

### 4.2. SlTRY Gene Isolation and Sequencing 

The promoter and protein-coding sequence of the *SlTRY* gene (XM_010328616) was amplified bia RT-PCR using RNA samples isolated from *S. lycopersicum* leaves. To obtain the sequence of the intron region of the *SlTRY* gene, we amplified the sequence from *S. lycopersicum* DNA. The primers used in this work are listed in Table S1. The RT-PCRs were conducted using a Bis-M1105 Thermal Cycler (Bis-N, Novosibirsk, Russia). All obtained RT-PCR products were cloned into pJET1.2/blunt vectors and sequenced as described in [45].

### 4.3. dsRNA Synthesis and Application

All dsRNAs used in this work were synthesized using the T7 RiboMAX™Express RNAi System (Promega, Madison, WI, USA). Four different dsRNA sequences were used in the work. The first dsRNA construct targeted only the promoter region of the *SlTRY* gene (dsRNA-Prom1, 308 bp). The second dsRNA construct targeted the promoter region and the initial part of the protein-coding sequence of the *SlTRY* gene (dsRNA-Prom2, 386 bp). The third dsRNA construct targeted the protein coding sequence of the *SlTRY* gene (dsRNA-TRY, 285 bp). The fourth dsRNA construct targeted the intron of the *SlTRY* gene (dsRNA-Intron, 483 bp).

All sequences were amplified through PCR to enable in vitro transcription and the production of dsRNA. A T7 promoter was incorporated at both the 5′ and 3′ termini of each sequence type using the PCRs and primers detailed in Table S1. The PCR reactions took place in the Bis-M1105 Thermal Cycler, with the program set according to the specifications of the T7 RiboMAX™ Express RNAi System. The resultant PCR products served as templates for transcription and dsRNA synthesis, following the manufacturer’s guidelines. To evaluate their purity, integrity and concentration, the produced dsRNAs were analyzed through gel electrophoresis and spectrophotometry. The Prom1-, Prom2-, Intron- and TRY-dsRNAs were administered to individual five-week-old *S. lycopersicum* plants using soft brushes, as described in [38].

For each dsRNA application, 70 μg of the dsRNA was dissolved in 400 μL of nuclease-free water and then sprayed onto the foliage (both the upper and lower surfaces of all tomato leaves were treated for each condition). In a separate experiment, two *S. lycopersicum* plants were utilized for each treatment type, specifically, two received 400 μL of sterile filtered water, while two others were administered the dsRNA of each variant. Next, the treated plants were divided into two groups for incubation: one under control conditions (22 °C, 16 h light) and the other under anthocyanin-inducing conditions (12 °C, 23 h light), maintained for seven days (Figure 2). Each type of analysis was conducted across at least four independent experiments. In all experiments, the dsRNAs were applied to five-week-old *S. lycopersicum* plants during late evening (21:00–21:30) under low-soil-moisture conditions. These specific conditions (appropriate plant age, late-evening timing, and low soil moisture) were identified as crucial for effective gene silencing in *A. thaliana* based on our recent analysis [29].

### 4.4. RNA Isolation and Reverse Transcription Reaction

For RNA extraction, one leaf of tomato was collected from a plant either from a (1) water application or before dsRNA and (2) seven days post treatment in a separate experiment. Total RNA was extracted as described in [46]. cDNAs were synthesized using the MMLV RT Kit (Evrogen, Moscow, Russia). The reactions were conducted in 40 μL of the reaction mixture, which included the first strand buffer, 4 μL of dNTP mix (10 mM each), 1.5 μL of oligo-(dT)15 primer (100 μM), 4 μL of DTT (dithiothreitol, 20 mM) and 3.4 μL of MMLV reverse transcriptase (100 u/μL) at 37 °C for 80 min. The resulting products were then amplified by PCR and checked for DNA contamination using primers for the *Actin* gene (NM_001330119.1).

### 4.5. Gene Expression Analysis by qRT–PCR 

The qRT-PCRs were conducted using SYBR Green I dye and a real-time PCR kit following the manufacturer’s instructions (Evrogen, Moscow, Russia). The process was carried out in a thermocycler equipped with a real-time PCR detection system (DNA Technology, Moscow, Russia) as described in [47]. Two internal control genes were used to normalize the data (*SlActin* (NM_001330119.1) and *SlUBI* (NM_001366381.1)). The expression was calculated with the 2−∆∆CT method [48]. The average values of gene expression before dsRNA treatment were taken as a unit.

### 4.6. Analysis of Secondary Metabolites

For HPLC-MS analysis, 100 mg of tomato leaves were frozen at −20 °C and homogenized using a mortar and a pestle. Shredded tissue was weighed and extracted for one day at 4 °C in 2 mL of 1% (*v*/*v*) HCl in methanol. Then, the mixture was centrifuged at 12,500 rpm for 10 min. The samples were passed through a nylon membrane with a pore size of 0.25 µm for further analysis. Secondary metabolites were identified using a 1260 Infinity analytical HPLC system (Agilent Technologies, Santa Clara, CA, USA) connected to aBruker HCT ultra PTM Discovery System (Bruker Daltonik GmbH, Bremen, Germany). The data were acquired in a positive and negative ion mode under the operating conditions as described in [49]. The MS spectra were recorded across an *m*/*z* range of 100–1500. HPLC with diode array detection (HPLC–DAD) for the quantification of all compounds was performed using an HPLC LC-20AD XR analytical system (Shimadzu, Kyoto, Japan). DAD data were collected in the 200–600 nm range. For the quantitative determination of anthocyanins, chromatograms were acquired at 530 nm, and for the remaining analyzed compounds, at 325 nm. The chromatographic separation was performed on a Shim-pack GIST C18 column (Shimadzu, Kyoto, Japan). The compounds were separated in a gradient of 0.1% formic acid (A) and acetonitrile (B). The elution profile was as follows: 0 to 35 min 0% of B; 35 to 40 min 40% of B; 40 to 50 min 50% of B; 50 to 65 min 100% of B. In total, 3 μL of the sample was injected at a constant column temperature of 40 °C and a flow rate of 0.2 mL/min.

The contents of anthocyanins were determined with external standard methods using the four-point regression calibration curves built with the available standards. The analytical standards—cyanidin chloride, petunidin chloride, delphinidin chloride, malvidin chloride, rutin, chlorogenic acid, caffeic acid and ferulic acid—were obtained from Sigma-Aldrich (St. Louis, MO, USA). All solvents were of HPLC grade.

### 4.7. Statistical Analysis

The data, presented as mean ± standard error (SE), were subjected to a one-way analysis of variance (ANOVA) followed by Tukey’s pairwise comparison test. The 0.05 level was selected as the point of minimal statistical significance. For each type of analysis, at least three independent experiments were performed, each with several technical replicates.

## Data Availability

The data presented in this study are available within the article and Supplementary Materials.

## Supplementary Materials

The following supporting information can be downloaded at https://www.mdpi.com/article/10.3390/plants13172489/s1: Table S1: Primers used in RT-PCR and qRT-PCR; Table S2: The content of anthocyanins in the leaves of *S. lycopersicum* grown under the control and anthocyanin-inducing conditions.
