# Supplementary material for: Regulation of Anthocyanin Accumulation in Tomato Solanum lycopersicum L. by Exogenous Synthetic dsRNA Targeting Different Regions of SlTRY Gene
Source: Plants (Basel). 2024 Sep 5;13(17):2489. doi: 10.3390/plants13172489 (PMC11396968; doi:10.3390/plants13172489)
Supplement: Supplementary file 1 [file plants-13-02489-s001.zip › plants-3182185-supplementary.pdf]

Table S1. Primers used in RT-PCR and qRT-PCR.

Abbreviation	Gene name	NCBI Gene ID	Forward primer, 5′-3′	Reverse primer, 5′-3′
FLS–reals	Flavonol synthase/flavanone 3-hydroxylase	Gene ID: 101249699	AGGTAGCAAGGGTCCAAGCA	GCTGCAGGCTGTTCGTTCTC
F3'H–reals	Flavonoid 3'-monooxygenase	Gene ID: 101266618	GGGCCATTGATGCACCTTCG	TGGGCGGCTCGAGAAGTTAG
RT–reals	Flavonol-3-O-glucoside L-rhamnosyltransferase	Gene ID: 101244316	CCACCTGCATTTCCTCACACC	CAGACCTGAGAGTACGCGGT
C3'H–reals	p-coumaroyl quinate/shikimate 3'-hydroxylase	Gene ID: 101246092	AACTCCATGGCCGGTAGTCG	TCGACCCGAACCAAACCGAA
4CL–reals	4-coumarate--CoA ligase	Gene ID: 101245039	CTGGGGCAGCACCATTAGGA	TTACTGTGCCACATGCCCCT
CHI–reals	Chalcone--flavonone isomerase	Gene ID: 101266223	GGGCAAAACAGGGGCAGAAC	AGCACTCTCTAGCTGCACACC
F3H–reals	Flavanone 3-dioxygenase	Gene ID: 100736482	GGTGGCAAGAAAGGTGGCTTC	CCAGCCTTGTGGTTTGTCTGG
CHS1 –reals	Chalcone synthase	Gene ID: 778294	ACTCGTCTCAGCAGCCCAAACTC	AAGCCCAACCTCACGTAGGTGTCC
CHS2 –reals	Chalcone synthase	Gene ID: 778295	AGAAGCAGCCCAAAAGGCCATTAAAG	CGGATAACGGTCCCACCAGCAAAG
ANS–reals	Anthocyanidin synthase	Gene ID: 101251607	TCTGGCCTAAAACCCCTGCTGAC	TCCTTCCTCCAATCCCAACCCAATC
TRY –reals	Transcription factor TRY	Gene ID: 104649435	GTGATGGGTTTGCACACAAGAG	GGGTCCCCAAACAAAATGGAAAA
Actin–reals	Actin 4	Gene ID: 101260631	GAA ATA GCA TAA GAT GGC AGA CG	ATA CCC ACC ATC ACA CCA GTA T
UBI–reals	Polyubiquitin	Gene ID: 101258282	GGA CGG ACG TAC TCT AGC TGA T	AGCTTTCGACCTCAAGGG TA
TRY	Transcription factor TRY	Gene ID: 104649435	ATGGATCAAAATCTCCATCA	TTATGTAGGTGGTAGACTTT
SITRY - prom1-dsRNA	Transcription factor TRY	Gene ID: 104649435	TAATACGACTCACTATAGGGAGAGAACTTTAACTATCCACTGG	TAATACGACTCACTATAGGGAGAGATCCATTTTTAGGTACAAAAGAA
SITRY – prom2-dsRNA	Transcription factor TRY	Gene ID: 104649435	TAATACGACTCACTATAGGGAGATGCCTAATGTGGAGAAACAAG	TAATACGACTCACTATAGGGAGACGGTGATGGAGATTTTGATCC
SITRY – intr-dsRNA	Transcription factor TRY	Gene ID: 104649435	TAATACGACTCACTATAGGGAGAGGAGACAGGTAAAAAAAAAGTTG	TAATACGACTCACTATAGGGAGACTTATATTCAAAAAATCATAAACTTAAG
SITRY–prom1	Transcription factor TRY	Gene ID: 104649435	GAACTTTAACTATCCACTGG	ATTTTTAGGTACAAAAGAA
SITRY–prom2	Transcription factor TRY	Gene ID: 104649435	TGCCTAATGTGGAGAAACAAG	CGGTGATGGAGATTTTGATCC
SITRY–intr	Transcription factor TRY	Gene ID: 104649435	GGAGACAGGTAAAAAAAAAGTTG	CTTATATTCAAAAAATCATAAACTTAAG

Table S2. The content of anthocyanins in mg per g of fresh weight (mg/g FW) in the leaves of *Solanum lycopersicum* grown under the control (+22 °C. 16 h light) and anthocyanin-inducing (+12 °C. 23 h light) conditions. Water — S. lycopersicum treated with sterile water; ds-Prom1 — S. lycopersicum treated with dsRNA-Prom1; ds-Prom2 — S. lycopersicum treated with dsRNA-Prom2; ds-Intron— S. lycopersicum treated with dsRNA-Intron; dsTRY — S. lycopersicum treated with dsRNA-TRY. The data are presented as the mean ± SE (three independent experiments). Means followed by the same letter in one row were not different using Student's t test. $p < 0.05$ was considered to be statistically significant.

№	Compounds	22-Water	22-ds-Intron	22-ds-Prom1	22-ds-Prom2	22-ds-TRY	12-Water	12-ds-Intron	12-ds-Prom1	12-ds-Prom2	12-ds-TRY
1	petunidin-3.5-O-diglucoside	0.013±0.009	0.015±0.011	0.059±0.021	0.056±0.018	0.172±0.035	0.093±0.039	0.089±0.04	0.255±0.071	0.547±0.135	0.381±0.101
2	petunidin-3-(caffeoyl)-rutinoside-5-glucoside	0.107±0.027	0.096±0.039	0.08±0.043	0.142±0.054	0.266±0.115	0.252±0.90	0.382±0.108	0.197±0.056	0.689±0.115	1.445±0.107
3	petunidin-3-(p-coumaroyl)-rutinoside-5-glucoside	0.312±0.060	0.245±0.069	0.403±0.104	1.076±0.191	1.52±0.291	0.832±0.052	0.982±0.156	1.083±0.178	1.535±0.116	3.801±0.458
4	delphinidin-3-O-(6"-O-p-coumaroyl)-glucoside	0.061±0.026	0.069±0.030	0.09±0.054	0.032±0.014	0.083±0.029	0.14±0.041	0.186±0.018	0.181±0.02	0.592±0.247	0.368±0.082
5	delphinidin-3-O-glucoside	0.031±0.008	0.023±0.010	0.051±0.021	0.02±0.006	0.059±0.014	0.07±0.047	0.015±0.007	0.013±0.005	0.084±0.035	0.039±0.012
6	malvidin-3-(p-coumaroyl)-rutinoside-5-glucoside	0.013±0.007	0.009±0.004	0.024±0.007	0.017±0.006	0.04±0.013	0.038±0.013	0.047±0.014	0.077±0.013	0.064±0.028	0.093±0.019
7	cyanidin-3-O-(6"-O-p-coumaroyl)-glucoside	0.002±0.002	0.002±0.002	0.011±0.007	0.002±0.001	0.008±0.003	0.01±0.005	0.013±0.006	0.029±0.008	0.038±0.012	0.039±0.01
	**Total**	**0.54±0.068**	**0.462±0.101**	**0.722±0.146**	**1.344±0.206**	**1.881±0.464**	**1.436±0.175**	**1.716±0.266**	**1.837±0.225**	**3.55±0.133**	**6.203±0.72**
